# Multiple Genomic Recombination Events in the Evolution of Saffold Cardiovirus

**DOI:** 10.1371/journal.pone.0074947

**Published:** 2013-09-23

**Authors:** Lili Ren, Yan Xiao, Jianguo Li, Lan Chen, Jing Zhang, Guy Vernet, Jianwei Wang

**Affiliations:** 1 MOH Key Laboratory of Systems Biology of Pathogens and Dr. Christophe Mérieux Laboratory, CAMS-Fondation Mérieux, Institute of Pathogen Biology (IPB), Chinese Academy of Medical Sciences (CAMS) and Peking Union Medical College, Beijing, P. R. China; 2 Fondation Mérieux, Lyon, France; University of Nebraska – Lincoln, United States of America

## Abstract

**Background:**

Saffold cardiovirus (SAFV) is a new human cardiovirus with 11 identified genotypes. Little is known about the natural history and pathogenicity of SAFVs.

**Methodology/Principal Findings:**

We sequenced the genome of five SAFV-1 strains which were identified from fecal samples taken from children with viral diarrhea in Beijing, China between March 2006 and November 2007, and analyzed the phylogenetic and phylodynamic properties of SAFVs using the genome sequences of every known SAFV genotypes. We identified multiple recombination events in our SAFV-1 strains, specifically recombination between SAFV-2, -3, -4, -9, -10 and the prototype SAFV-1 strain in the VP4 region and recombination between SAFV-4, -6, -8, -10, -11 and prototype SAFV-1 in the VP1/2A region. Notably, recombination in the structural gene VP4 is a rare event in Cardiovirus. The ratio of nonsynonymous substitutions to synonymous substitutions indicates a purifying selection of the SAFV genome. Phylogenetic and molecular clock analysis indicates the existence of at least two subclades of SAFV-1 with different origins. Subclade 1 includes two strains isolated from Pakistan, whereas subclade 2 includes the prototype strain and strains isolated in China, Pakistan, and Afghanistan. The most recent common ancestor of all SAFV genotypes dates to the 1710s, and SAFV-1, -2, and -3 to the 1940s, 1950s, and 1960s, respectively. No obvious relationship between variation and pathogenicity exists in the critical domains of the CD and EF loops of viral capsid proteins or the multi-functional proteins L based on animo acid sequence identity comparison between SAFV genotypes.

**Conclusions/Significance:**

Our findings suggest that intertypic recombination plays an important role in the diversity of SAFVs, highlighting the diversity of the five strains with the previously described SAFV-1 strains.

## Introduction

The newly identified Saffold cardiovirus (SAFV) is a member of the Cardiovirus genus and belongs to the *Picornaviridae* family [1]. SAFV is the member of the *Theilovirus* species, which includes Theiler’s murine encephalomyelitis virus (TMEV), Theiler-like rat virus (TRV), and Vilyuisk human encephalomyelitis virus (VHEV) [1]–[3]. Similar to the genomes of other cardioviruses, the SAFV genome is a positive, single-stranded RNA of approximately 8,050 nucleotides (nts). The genome contains a single reading frame, encoding the leader (L) protein, four capsid proteins (VP1 to VP4), and seven nonstructural proteins (2A, 2B, 2C, 3A, 3B, 3C and 3D) [1]. Unlike other members of the Theilovirus and Encephalomyocarditis virus (EMCV) species, SAFV shows high genetic diversity [1], [4]–[7].

The prototype strain of SAFV, designated as SAFV genotype 1 (SAFV-1), was identified in 2007 using genome sequencing [1]. It was first isolated in 1981, from a female child with fever of unknown origin in California, USA [1]. Since then, SAFVs have been identified in human stool and respiratory samples, and 11 genotypes have been documented based on phylogenetic analysis of the VP1 gene [1], [8]. A large-scale serological survey indicated that SAFV infection occurs early in life [9]. The wide detection of SAFVs in humans and serological study indicates that SAFVs are authentic human cardioviruses.

Etiological studies have shown the difference in geographical distribution of SAFV genotypes. SAFV-2 and -3 are the predominant genotypes found in stool and respiratory specimens in America, Asia, and Europe [8]; whereas SAFV-4-11 are primarily found in stool samples from south Asia (Pakistan and Afghanistan) [4]. Although SAFV-1 was the first SAFV strain identified, it was not reported again until 2009, when a SAFV-1 epidemic was discovered in stool and respiratory samples from patients in China [10]–[12]. More recently, SAFV-1 has been isolated in samples from Pakistan and Afghanistan. Analysis of the SAFV genomes has identified recombination events between different SAFV genotypes. For example, recombination between the SAFV-5 strain Pak5152 and the SAFV-6 strain Pak6572 indicates that different genotypes interact during the transmission and spread of SAFVs [4], [13], [14].

Although SAFV is the first human cardiovirus identified, little is known about its natural history and it is unclear why there was a 20-year hiatus between its first isolation and subsequent detection. A powerful tool for tracing the origin and evolution of a virus is phylogenetic and phylodynamic analysis of viral sequences. Data obtained using such methods have contributed to the surveillance of viral spread and drug resistance as well as the identification of strains as vaccine candidates [15], [16]. However, the analysis of the SAFV genome is still insufficient and existing data has been based on very limited SAFV sequences that do not include all known SAFV genotypes. Genomic and phylodynamic analysis based on all available genotypes with updated SAFV genome sequences is needed to provide insight into the natural history and pathogenicity of SAFV.

In this study, we employed phylogenetic and phylodynamic analysis using five additional SAFV-1 near-full-length genome sequences identified together with previously reported sequences of SAFV 1-11 to analyze the phylogenetic relationship as well as genome features of SAFV. We also used the VP1 sequences to estimate the divergence time and evolution rate of SAFV.

## Results

### Genome sequencing of SAFV-1 strains

To characterize the SAFV genomes, five near full-length genomes of SAFV-1 strains (BCHGL352, BCHGL362, BCHGL365, BCHGL368 and BCHGL371) were amplified and sequenced. The genome sequences include the entire coding regions of L, P1, P2, and P3 genes as well as the 3’UTR and partial 5’UTR genes, for a total length of 7,851 to 7,868 nts. The G+C content was 43.2%. These five genomes were aligned with other full-length or near-full-length genomes of SAFVs using clustal W implemented in Mega 4.0, and the 7,341nt fragment (corresponding to nt 290 to 7,360 of reference strain NC009448) was used for further analysis. We found that the five strains share nucleotide (nt) identities of 99.6–99.8% and amino acid (aa) identities of 99.3-99.7% between each other, and 88.9% and 97–97.2% nucleotide and aa identities with the prototype SAFV-1 strain (NC009448), respectively (Table 1). The SAFV-1 strains share less than 81% aa identity with the other genotypes in the VP1 gene region. However, the amino acid sequences of the five SAFV-1 strains were 100% identical to several strains of SAFV-2, -3, -4 and -10 in the VP4 gene and more than 94% identical in the P2 and P3 genes.

**Table 1:** Pair-wise sequence identity between amino acids of SAFV-1 genotypes identified in this study, prototype SAFV-1, SAFV-1 Pak3079, and other genotypes.

	% identity^b^															
	SAFV-1						SAFV-2	SAFV-3	SAFV-4	SAFV-5	SAFV-6	SAFV-7	SAFV-8	SAFV-9	SAFV-10	SAFV-11
Region	BCHGL362	BCHGL365	BCHGL368	BCHGL371	NC009448	Pak3097										
5'-UTR a	99.8	100	99.8	99.8	94.6–94.9	95.0–95.3	92.4–96.4	90.9–94.9	95.3–95.6	92.9–94.9	94.4–94.6	93.4–93.6	93.2–93.4	95.6–95.8	93.3–93.6	94.4–94.6
Polyprotein	99.4	99.5	99.4	99.2	97.0–97.2	97.8–98.1	90.0–91.8	89.2–89.9	92.5–92.8	88.9–89.4	88.4–89.3	89.6–90.0	89.1–89.5	89.4–89.8	89.2–89.6	89.0–89.4
Leader protein	100	100	100	100	84.5	84.5	78.8–95.7	84.5–94.3	91.5	85.9–87.3	80.2–81.6	78.8	87.3	90.1	92.9	85.9
P1	100	99.8	100	99.8	96.6–96.7	97.3–97.4	80.3–82.3	77.1–77.9	84.0–84.1	75.5–75.8	76.0–76.3	67.4–67.6	76.8–77.0	76.8–77.0	76.8–77.0	76.7–76.8
VP4	100	100	100	100	97.2	98.6	88.8–100	97.2–100	100	98.6	98.6	94.4	97.2	98.6	100	97.2
VP2	100	100	100	99.6	96.2–96.6	98.5–98.8	82.8–84.3	77.8–78.9	82.8–83.2	79.7–80.4	77.4–78.2	78.5–78.9	76.0–76.3	78.2–78.5	77.8–78.2	77.8–78.2
VP3	100	99.5	100	100	98.2–98.6	99.1–99.5	84.3–84.7	84.8–86.5	89.1–89.5	77.1–78.4	81.0–81.4	87.0–87.4	85.2–85.7	84.8–85.2	85.7–86.1	84.4–84.8
VP1	100	100	100	100	98.9	98.5	74.9–76.3	66.1–67.2	81.0	67.0–68.1	65.2–66.6	68.3	66.1	63.6	63.2	64.7
P2	98.9	98.9	98.4	98.4	96.5–97.6	97.4–98.4	94.3–98.8	95.8–97.9	96.9–97.9	96.7–98.1	93.9–98.2	97.2–98.2	97.2–98.2	97.9–98.9	96.7–97.7	96.9–97.9
2A	100	100	100	98.5	96.4–97.8	98.5–100	95.0–100	94.3–97.8	95.7–97.1	95.7–97.1	91.5–98.5	96.4–97.8	97.1–98.5	97.8–99.2	95.7–97.1	97.8–99.2
2B	100	99.2	100	100	97.6–98.4	97.6–98.4	92.9–99.2	96.0–98.4	98.4–99.2	97.6–98.4	96.8–98.4	97.6–98.4	97.6–98.4	97.6–98.4	96.8–97.6	96.0–96.8
2C	98.1	98.4	97.1	97.7	95.2–97.4	95.8–98.1	93.3–99.0	94.6–98.4	95.8–98.1	95.8–98.7	95.5–98.4	96.5–98.7	96.2–98.4	97.1–99.3	96.2–98.4	95.8–98.1
P3	99.2	99.5	99.5	99.1	97.0–97.5	98.0–98.5	96.1–98.2	96.3–98.2	97.3–97.8	96.9–97.9	97.5–98.2	97.3–97.8	96.9–97.3	97.0–97.5	96.9–97.3	97.2–97.6
3AB	100	100	100	100	98.1	99.0	98.1–100	97.1–99.0	98.1	98.1–99.0	98.1–99.0	98.1	99.0	99.0	98.1	99.0
3C	99.0	100	100	100	98.1	98.1–98.6	94.4–98.6	95.8–98.6	98.6–99.0	97.2–98.1	98.6–99.0	98.6–99.0	96.7–97.2	97.2–97.6	98.1–98.6	98.1–98.6
3D	99.1	99.1	99.1	98.3	96.9–97.7	97.5–98.3	95.5–98.3	95.2–97.7	96.1–96.9	95.5–97.5	96.3–97.5	96.1–96.9	96.1–96.9	96.1–96.9	95.5–96.3	95.8–96.6

a Nucleic acid sequence identities between the SAFV-1 strains described here (BCHGL352 as query strain) and prototype SAFV-1, SAFV-1 Pak3079 and other genotypes.

b The identities listed in NC009448, Pak3097, SAFV-2-11 are calculated between all the five strains identified in this study and the according strains.

### Phylogenetic analysis

**Figure 1:**
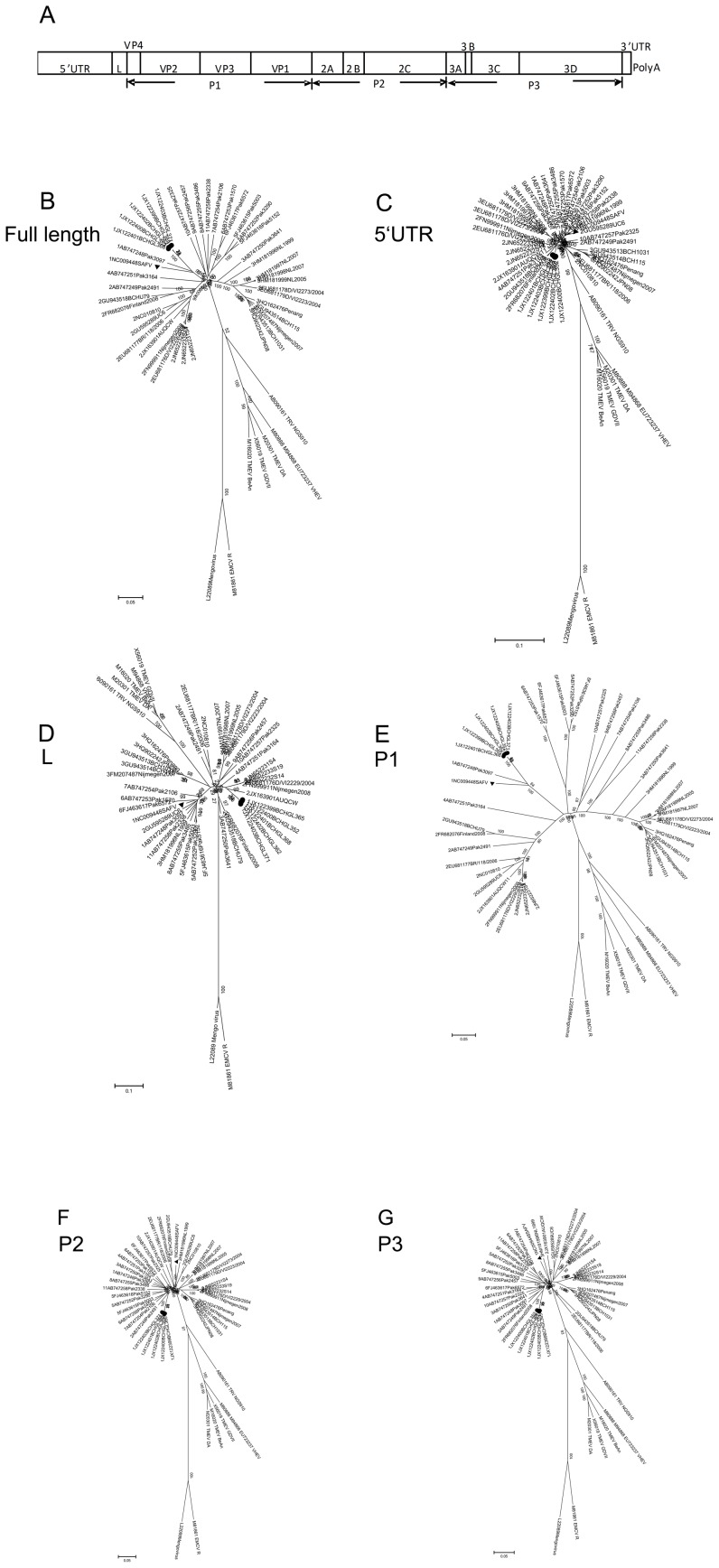
Phylogenetic analysis of different SAFV genotypes. Genome map of SAFVs (A). The trees of complete genomes (B), 5′-UTR (C), L(D), P1 (E), P2 (F), and P3 (G) regions were constructed using the neighbor-joining method. Triangles represent the prototype SAFV-1 strain. Circles represent SAFV-1 strains identified in this study.

To address the genetic relationships among different genotypes further, we carried out phylogenetic analysis of the full-length genomes and the P1 region of SAFVs. The results confirm that our five SAFV-1 strains are clustered with the prototype SAFV-1 (NC009448) (Fig. 1). The results of the analysis on the individual regions, including VP4, VP2, VP3 and VP1, also support this conclusion (data not shown).

However, in the cases of the 5’UTRs, the L protein, and the P2 and the P3 regions, the five SAFV-1 strains were separated from the prototype strain of SAFV-1 in the phylogenetic tree, forming a different lineage (Fig. 1). The results of the phylogenetic analysis of the individual nonstructural genes (2A, 2B, 2C, 3AB, 3C and 3D) were consistent with those of the P2 and P3 regions (data not shown). Taken together, these data indicate that our SAFV-1 strains are genetically distinct from the prototype strain.

**Figure 2:**
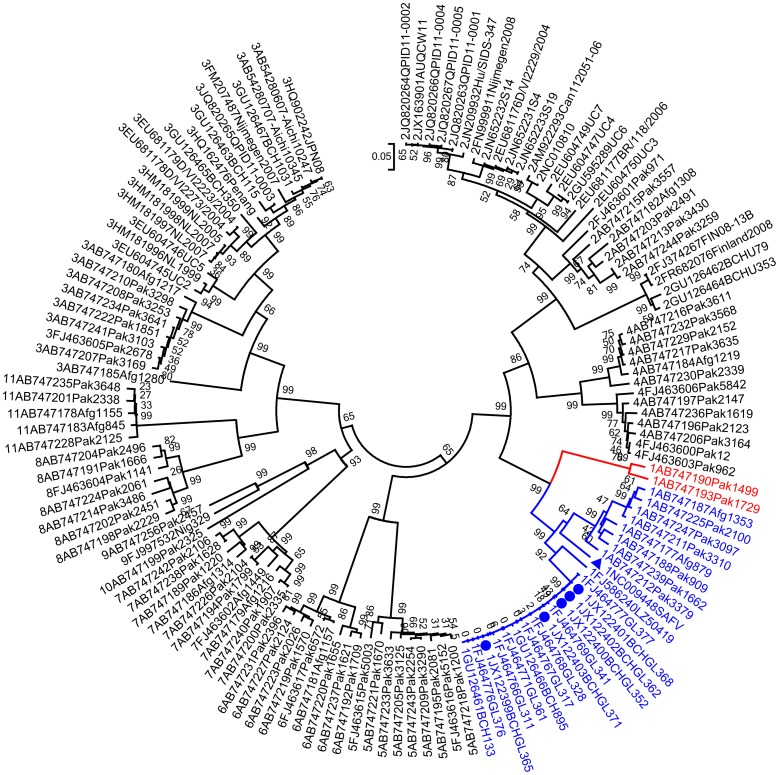
Phylogenetic analysis of the VP1 gene sequence of SAFVs. The trees were constructed using the neighbor-joining method. Each strain is labeled by number of genotype, accession number and strain name. Genotype I (SAFV-1) is highlighted in color, sublade 1 in blue, and subclade 2 in red. The triangle represents the prototype SAFV-1 strain. Circles represent the SAFV-1 strains identified in this study.

As VP1 gene is responsible for genotype classification in Picornaviruses [4], to investigate the diversity of SAFV-1, we used our SAFV-1 strains and all of the SAFV VP1 sequences available in GenBank to construct a phylogenetic tree (Fig. 2). It shows that at least two distinct subclades exist within SAFV-1 strains. Subclade 1 (in red) includes only two strains, which were isolated from Pakistan. Most SAFV-1 strains identified belong to subclade 2 (in blue), which includes the prototype strain, the strains identified by our group, the Pakistan strains, the Afghanistan strains and other strains identified in China by other groups. In subclade 2, the strains identified in China clustered together, while the prototype and the strains identified in Pakistan and Afghanistan clustered, indicating different evolution characteristics. The mean pairwise *p* distances of nt and aa in the two subclades are 0.22 and 0.073, respectively.

### Recombination analysis

**Figure 3:**
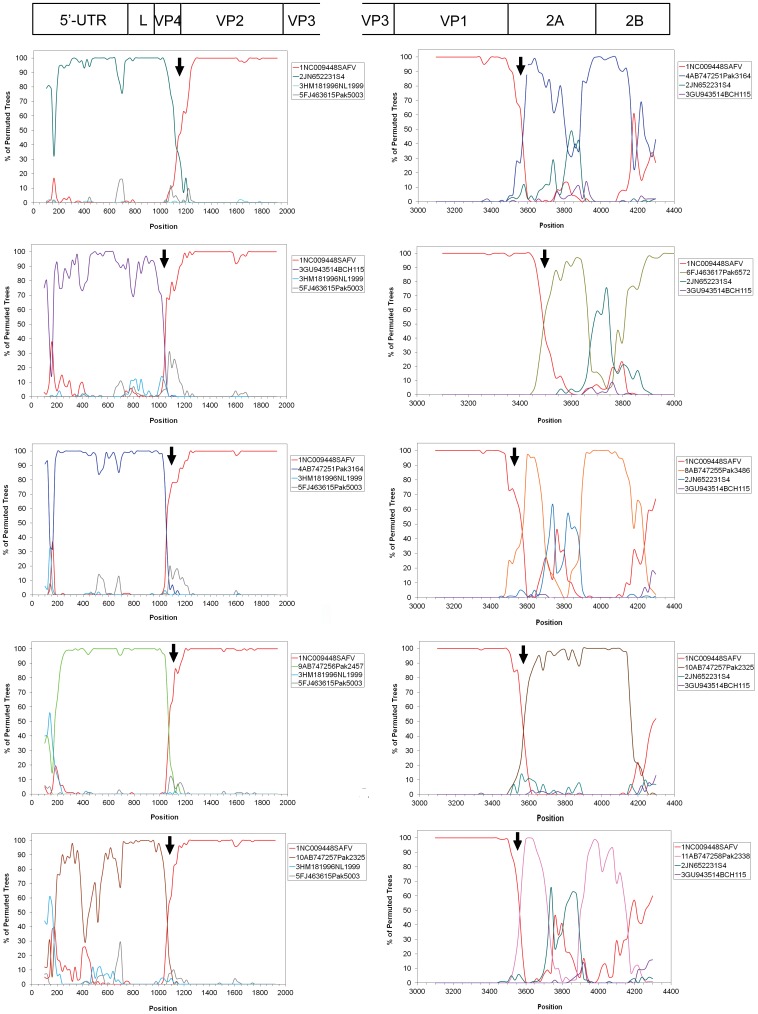
Recombination analyses of SAFV-1. Bootscanning plot analysis of the SAFV-1 sequence identified in this study in comparison to those of other genotypes. The graph generated by using BCHGL352 as representative query sequence and prototype strain sequence, a reference sequence positioned closely to the query sequence in the phylogenetic tree and two distantly related genomes were used as the outgroup sequences. The bootstrap value is 100 for a window of 200 bp. Arrows indicate putative recombination breakpoints.

The inconsistencies in the branching orders of the SAFV-1 clusters in the phylogenetic trees suggest that the SAFV-1 genotype was probably generated by a recombination event. To assess these potential recombination events, bootscanning-plot analyses were performed. For each bootscanning plot, the genome sequences of our SAFV-1 strain were used as query sequence. The prototype SAFV-1 sequence, two reference sequences positioned closely to the query sequence in the phylogenetic tree and a distantly related genome were used as the outgroup sequence. A representative result of bootscanning in which the BCHGL352 (JX122400) strain was used as a query sequence (Fig. 3), shows that in the regions of the 5′-UTR and L genes, BCHGL352 is most closely related to SAFV-2 (S4 strain, JN652231), SAFV-3 (BCH115 strain, GU943514), SAFV-4 (Pak3164 stain, AB747251), SAFV-9 (Pak2457 strain, AB747256), and SAFV-10 (Pak2325 stain, AB747257). Additionally, a potential recombination site was found in VP4 region. In the P1 region, BCGL352 was most closely related to the prototype SAFV-1 (NC009448). Around the boundary of the P1 and P2 gene regions, we found another potential recombination site between the prototype SAFV-1 (NC009448) and SAFV-4 (Pak3164 stain, AB747251), SAFV-6 (Pak6572 stain, FJ463617), SAFV-8 (Pak3486 stain, AB747255), SAFV-10 (Pak2325 stain, AB747257), and SAFV-11 (Pak2338 stain, AB747258). Similar bootscanning results were obtained when any one of the five SAFV-1 sequences was used as a query strain (data not shown).

The potential recombination events were further confirmed using the GARD program and the KH test. Several potential recombination breakpoints were found in the five SAFV-1 strains. According to the regions identified by the bootscanning results, statistically significant recombination breakpoints are located at nt 1,078 and nt 3,651 (*p* = 0.0006), nt 1,078 and nt 3,703 (*p* = 0.0006), nt 1,078 and nt 3,632 (*p* = 0.0006), nt 1,078 and nt 3,682 (*p* = 0.0006), nt 1,078 and nt 3,703 (*p* = 0.0006) in the genome when BCHGL352, 362, 365, 368, and 371 are used as query sequences, respectively. The recombination sites were mapped in the VP4 and 2A genome regions. However, neither the SAFV-2 strains of the BR/118/2006 (EU681177), HTMV (NC010810), and UC6 (GU595289) isolates from Germany and the US nor the SAFV-3 stains of Pak3641 (AB747250), BCH1031 (GU943513), and NL1999 (HM181996) isolates from Pakistan, China, and the Netherlands appear to be involved in the recombination of the VP4 gene region.

Taken together, these findings suggest that our five SAFV-1 strains might derive from complicated recombination events between the prototype strain and multiple genotypes that circulate in nature.

### Variability analysis

The functional domain in the capsid and the L protein play an important role in the persistence and pathogenicity of cardioviruses [8]. To determine if the described SAFV-1 strain has characteristics distinct from the prototype strains, other genotypes, and animal cardioviruses, we analyzed the main immunogenetic domains located in the viral capsid proteins, including the CD loop in VP1, the EF loop in VP2, and the L protein.

**Figure 4:**
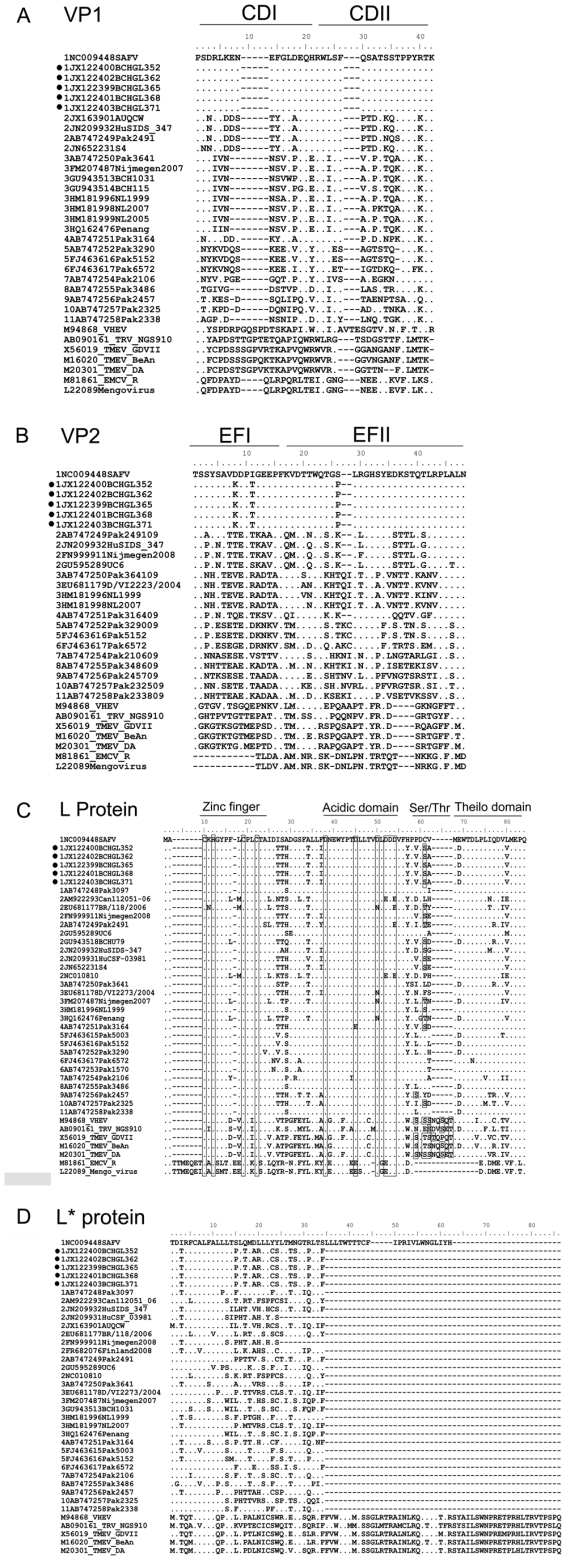
Alignment of amino acid sequences of the major surface structures among all the genotypes of SAFV, together with homologues from other theiloviruses and EMCVs, including the VP1 CD loop (A), VP2 EF loop (B), L protein (C), and L* protein (D). Periods indicate identity to the first sequence on the top, and dashes indicate amino acid deletions.

All the SAFV-1 strains identified in this study have the same sequence in the CDEF loops. In the CD loops, the aa sequence of the five SAFV-1 strains is 100% identical to that of the prototype strain and 29.4% to 100% among all known SAFVs and from 7.5% to 26.1% identical to that of animal cardioviruses. In the EF loops, the aa sequence of the five strains is 93% identical to that of the prototype strain, with aa sequence identity ranging from 35.4% to 100% among all known SAFVs and from 12.5% to 25.0% among animal cardioviruses (Fig. 4A and B).

The L protein of our SAFV-1 strains contains the conserved putative zinc finger domain and the acidic domain, but has one more serine amino acid in the Ser/Thr phosphorylation domain than the prototype SAFV-1 strain. The five SAFV-1 strains we identified also contain a sequence of ME/DWTD/NLP in the carboxy terminal, which is referred to as the Theilo domain and conserved in theiloviruses (Fig. 4C).

Studies have shown that the variations in the L protein may affect its pathogenesis [17]. Two strains (JN209931 and JN209932) were identified in aseptic samples from two patients with invasive SAFV infections, which were attributed to the viral pathogenesis of these strains [18]. To investigate the potential pathogenesis of our SAFV-1 strains, we compared the sequences of the functional domains or the L protein with the JN209931 and JN209932 strains. We found no significant difference between the aa in the four domains of these strains and the domains of other strains from different genotypes, indicating that the invasive SAFV strains might not be related to specific genome sequences in the functional domains.

In addition, compared with the prototype strain, which has a 57 aa L* protein, our SAFV-1 strains have a 34 aa L* protein translated from a putative opening reading frame initiated from ACG [19], whereas some strains of SAFV-2 have only a 24 aa L* protein (Fig. 4D).

### Evolutionary analysis of SAFV genotypes

To evaluate the selection pressure on the SAFV genes during evolution, we estimated the mean *dN/dS* values of all codons among branches of the SAFVs. We found that in the P1, P2, and P3 genome regions, the estimated values of mean *dN/dS* are 0.0473, 0.0346, and 0.0419, respectively for all genotypes, suggesting a purifying selection.

**Figure 5:**
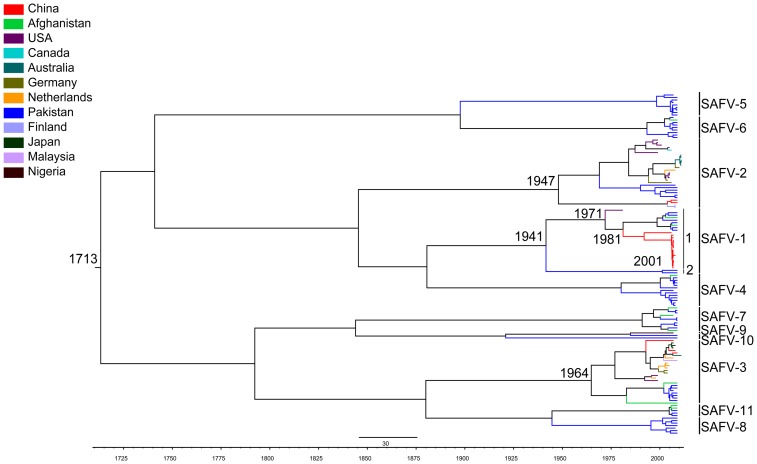
Estimated time at which the most recent common ancestor of SAFV genotypes emerged. The phylogeny of time-scale was summarized from MCMC phylogenies of the VP1 gene and the data was analyzed using a relaxed-clock model with uncorrelated exponential distribution in BEAST (V 1.6.1).

The Bayesian relaxed molecular clock method with uncorrelated lognormal distribution was used to infer the evolutionary behavior of SAFVs. The mean nucleotide substitution rate of the VP1 gene was calculated to be 3.63 (2.29–4.99)×10^−3^ substitutions/site/year for the SAFVs. Molecular clock analysis using the VP1 gene showed that the mean time of the most recent common ancestor (tMRCA) of all SAFVs was 1,694 (95% highest posterior density [HPDs], 1,568 to 1,835). The tMRCA of SAFV-1 was 1,941 (HPDs, 1,910 to 1,967); that of SAFV-2 was 1,947 (HPDs, 1,912 to 1,977); and that of SAFV-3 was 1,964 (HPDs, 1,938 to 1,985) (Fig. 5). The tMRCA of other SAFVs is described in the MCMC tree, but the analysis of the evolutionary rate and tMRCA may not be reliable as the number of VP1 gene sequences available for other genotypes is limited. Consistent with results of the phylogenetic analysis of the VP1 gene, results of the Bayesian MCMC analysis of the VP1 gene also suggest that at least two distinct subclades of SAFV-1 strains exist.

## Discussion

To assess the natural history of SAFVs, we sequenced the nearly complete genomes of five SAFV-1 strains and analyzed the phylogenetic and phylodynamic features of SAFVs using all the sequences of each SAFV genotype available in GenBank. Although all of the SAFV-1 strains have high identity in the P1 gene region, we found that our five SAFV-1 strains showed phylogenetic relationships distinct from those of the prototype stain of SAFV-1 in other gene regions. Bootscanning and GARD analysis showed that complicated recombination events occurred between the prototype SAFV-1 strain and SAFV-2, -3, -4, -9, and -10 in the VP4 gene region, and between the prototype SAFV-1 strain and SAFV-4, -6, -8, -10 and -11 in the 2A regions, leading to the generation of our SAFV-1 strains. However, the prototype strain of SAFV-1 does not have the same recombination profile as our SAFV-1 strains. This profile is also different from that observed in other SAFV-1 strains. In the genome of a SAFV-1 strain from Pakistan (Pak3097, AB747248), a potential recombination event was found in the L/VP4 region between several strains of SAFV-2, -4, -9 and the prototype strain, but no recombination was found in the VP1/2A region (data not shown). These data indicate that recombination may play an important role in the evolution of SAFV-1 strains (including those from China and Pakistan). However, we cannot exclude other natural recombination events among these genotypes as the whole genome sequences of SAFV-1 deposited in GenBank are very limited.

Viral recombination is a powerful contributor to genetic variation, adaptation to new hosts, escape from the host immune response, and emergence of newly infectious agents [20]–[24]. Genetic recombination has been documented and confirmed as a common phenomenon in the *Picornaviradae* genus, which includes enterovirus, apthovirus, parechovirus, cosavirus, and cardiovirus [4], [13], [14], [25]–[29]. The prototype SAFV-1 strain has been reported to be involved in a recombination event with SAFV-2 strain BR/118/2006, which led to the generation of SAFV-2 strains HTMV (NC010810), Can112051-06, and HTMV-UC6 [14]. We found that the prototype strain served as a parent strain to form the new strains of SAFV-1 by intertypic recombination with other genotypes. This finding is consistent with our molecular clock model using the VP1 gene sequences, which estimated he tMRCA of the prototype strain to be 1971, earlier than that of the recombinant strains. These data support our conclusion that the recombinant strains were derived from the prototype SAFV-1.

The role of recombination in SAFV-1 evolution is also supported by the results of natural selection analysis. The values of mean *dN/dS* ratios are very low, ranging from 0.0346 to 0.0473 in coding regions, although SAFV contains a high substitution rate similar to that of enteroviruses [30]. The low *dN/dS* ratios indicate a purifying selection pressure on the SAFV genome. Our results are consistent with a previous study, in which the *dN/dS* ratios of different SAFV genome regions were also very low [13]. These findings indicate an important role of inter- and intratypic recombination in driving SAFV evolution.

We found a rare intertypic recombination site located in the VP4 gene. No intertypic recombination sites in the structural gene region have been described for SAFV, although an intratypic recombination in the VP4-VP2 genome region between the SAFV-2 strain BR/118/2006 and strain D/VI2229/2004 has been reported [13]. Recombination in structural regions appears to be uncommon in picornaviruses, as recombination hot spots are usually found at the boundary between the structural and nonstructural regions or between nonstructural regions, such as between P2 and P3 or the 5′-UTR/L and VP1/2A [26], [31]–[33]. A few reports have described recombination events in the structural gene regions, for example, at the junction of the VP2/VP3 gene in the foot-and-mouth disease virus, between the VP4/VP2 and VP1 gene regions of coxsakie B1 virus, and in the VP1 gene region of TMEV [3], [13], [32], [34]. It is unclear if these complicated recombination events have occurred in different genotypes and if the recombination site in the VP4 gene region provides a selective advantage for virus diversification and evolution; the molecular mechanisms behind these recombination events also need to be clarified [35], [36].

Based on the results of phylogenetic analysis and MCMC tree construction of VP1, there are at least two subclades of SAFV-1. The high genetic diversity of SAFV-1 indicates a complicated or very different etiological process of SAFV-1. There is no apparent difference in geographical distribution of the two subclades. Additional sequences are needed to determine if there is a distinct sublineage of subclade 2.

Because the sequences of SAFVs are only available from a short period and the sequence numbers of some genotypes are limited, the phylodynamic features of SAFVs should be further addressed by the accumulation of genome sequences from different geographical locations and over a longer time-span. However, in our study, we identified complicated recombination in SAFVs through phylogenetic and phylodynamics analysis based on whole viral genome sequences. Additionally, we are the first to identify an uncommon recombinant site in the viral structural protein VP4. Our results reveal new phylogenetic relationships between SAFV genotypes and provide insight into the evolution of SAFVs.

## Materials and Methods

### Samples and sequencing

In our previous study, 12 SAFV-1 positive samples were identified in fecal samples from pediatric outpatients with gastroenteritis at the Beijing Children’s Hospital in November 2007 [10]. The patients were recruited for a prospective study on viral etiology of diarrhea between March 2006 and November 2007. Written informed consent was obtained from the next of kin, caretakers, or guardians on the behalf of the minor/children participants involved in this study. This study was approved by the ethical review committee of the Institute of Pathogen Biology, Chinese Academy of Medical Sciences.

Five near-full genome sequences were amplified from those samples. Viral nucleic acids were extracted using the NucliSens easyMAG platform (bioMérieux, Marcy l’Etoile, France), according to the manufacturer’s instructions. A combined random priming and oligo (dT) priming strategy was used to derive cDNA from an RNA template. The primer sets used to amplify the SAFV sequences were designed from multiple alignments of SAFV-1 (NC009448) with other genotypes of SAFV (Table 2), and the full genomic sequences were obtained using a genome walking method. PCR products were cloned into the pGEM-T Easy vector (Promega, Madison, WI, USA), and at least three positive clones were verified by sequence analysis. The entire sequences were assembled manually to produce final viral genome sequences.

**Table 2:** Primers used for the genome amplification of the saffold cardiovirus genotype I.

Primer name	Primer direction, sequence (5'-3')	Amplified gene
CF188^7^	Forward, CTAATCAGAGGAAAGTCAGCAT	5′-UTR
CR990^7^	Reverse, GACCACTTGGTTTGGAGAAGCT	
SAF720	Forward, AGCTGTAGCGACCTCACAATAGC	L, VP4, VP2
SAF1878	Reverse, GAAGTGTCAAATTCTGGAGCCAT	
SAF1857	Forward, TTATGGCTCCAGAATTTGACAC	VP2, VP3
SAF2446	Reverse, CCAAGAAAGGTTGGGATTTTACAT	
SAF2382	Reverse, TACATGTGCGGAGAATTCACTGA	VP3
SAF3023	Forward, GTCAGCTGATGCATTATCATCTGAAAC	
SAF2840	Forward, TCAGAATGCCAATCTCCCCAAC	VP1
SAF3718	Reverse, AAAGGTCCACCCGATACATTGA	
SAF3693	Forward, TGAGAGTBTTYTGYCCAAGRCCHA	2AB
SAF4497	Reverse, ATTCCAGCCATCAAAGAYAGACAGA	
SAF4433	Forward, TCAGTTCTTTACCTACATAATCCGGAC	2C
SAF5578	Reverse, TGATTGAGCRACYAATGTGTTCATT	
SAF5448	Forward, AGAGGCGTACGGTTTGTATGTAACC	3A, 3B, 3C
SAF6453	Reverse, AGTGCATTGCGTACACAGCCTTT	
SAF6363	Forward, TWCACTAYAGAGCYACAACACAYC	3D, 3′-UTR
SAF7395	Reverse, CTTCTYTCTTCAAAAGCATGTCG	
SAF7310	Forward, TTYACAMCAGAGAATGGATTTGAC	3′-UTR
SAF8051	Reverse, GTTCTCATTTCCAATTAAAAGCT	

### Sequence collection and phylogenetic analysis

All available SAFV sequences were retrieved from GenBank (www.ncbi.nlm.nih.gov) on 22 October 2012. A total of 42 near full-length genomes, including 37 genomes deposited in GenBank and 5 genomes from this study, and 136 partial VP1 genes from GenBank were subjected to phylogenetic analysis. The background information of all the sequences including accession numbers, collection dates, and isolation areas were provided in the supplemental materials (Table S1). The sequences were aligned using Clustal W and phylogenetic trees were constructed using the neighbor-joining (NJ) method with Kimura’s two-parameter model and 1000 bootstrap pseudo-replicates implemented in the MEGA 4.0 [37]. The pair-wise sequence identities in each region were calculated for the comparison of sequence divergence using BioEdit. The mean pairwise *p* distances of nucleotide and animo acid sequences in VP1 gene were calculated using MEGA 4.0 [37].

### Recombination analysis

To analyze the recombination events, genomes of SAFV were aligned and analyzed using SimPlot (V3.5.1, thhp://sray.med.som.jhmi.edu/SCRoftware). The bootscanning method was used in SimPlot to assess the potential recombination relationships. The window and step sizes were determined according to the length of the sequences. Recombination sites were confirmed using the Kishino-Hasegawa (KH) test in the Genetic Algorithms for Recombination Detection (GARD) program implemented in the HYPHY package accessed through the DataMonkey facility (http://www.datamonkey.org) [38]. The best-fit nucleotide substitution model for the datasets used in this study was determined using ModelTest (v 3.7) [39].

### Natural selection and adaption analysis

Using the codon-based phylogenetic method implemented in CODEML (distributed in PAML, version 4) [40], the selection pressure of each coding gene of SAFV was investigated through estimating the ratio of nonsynonymous to synonymous substitutions (*dN/dS*).

### Bayesian MCMC evolutionary analysis

The Bayesian Markov Chain Monte Carlo (Bayesian MCMC) method implemented in BEAST (v1.6.1) was used to determine the divergence time, rates of evolution, and molecular clock phylogenies [41]. Datasets of the VP1 gene were constructed to perform the Bayesian phylogenetic analysis. Based on the results of the ModelTest, the best-fit model of evolution was used under the GTR substitution model with a gamma distribution ([GTR+G]). For the Bayesian MCMC, there were 500 million generations with sampling every 1,000 generations using a relaxed molecular clock model (the uncorrelated lognormal distributed model). Constant population size and Bayesian skyline distribution were adopted for making inferences. The resulting tree of each run was annotated using Tree Annotator, implemented in Beast (v1.6.1) and the maximum clade credibility tree was visualized using Figtree software (v1.1.2).

### Nucleotide sequence accession numbers

The five near-full-length nucleotide sequences determined in this study were deposited in GenBank and assigned accession numbers JX122399, JX122340, JX122341, JX122342, and JX122343.

## Supporting Information

Table S1 **Background information of the Saffold cardiovirus (SAFV) gene sequences used in this study.**
(DOC)
