# Supplementary material for: Multiple Genomic Recombination Events in the Evolution of Saffold Cardiovirus
Source: PLoS One. 2013 Sep 23;8(9):e74947. doi: 10.1371/journal.pone.0074947 (PMC3781130; doi:10.1371/journal.pone.0074947)
Supplement: Table S1 — Background information of the Saffold cardiovirus (SAFV) gene sequences used in this study. (DOC) [file pone.0074947.s001.doc]

Table S1. Background information of the Saffold cardiovirus (SAFV) gene sequences used in this study.

| Virus | strain | Collection date | Isolation area | Accession no. | Gene |
| --- | --- | --- | --- | --- | --- |
|
| SAFV-1 | SAFV | 1981 | California, USA | NC009448 | genome |
|  | BCHGL352 | 2007 | Beijing, China | JX122400 | genome |
|  | BCHGL362 | 2007 | Beijing, China | JX122402 | genome |
|  | BCHGL365 | 2007 | Beijing, China | JX122399 | genome |
|  | BCHGL368 | 2007 | Beijing, China | JX122401 | genome |
|  | BCHGL371 | 2007 | Beijing, China | JX122403 | genome |
|  | GL311 | 2007 | Beijing, China | FJ464766 | VP1 |
|  | GL317 | 2007 | Beijing, China | FJ464767 | VP1 |
|  | GL328 | 2007 | Beijing, China | FJ464768 | VP1 |
|  | GL341 | 2007 | Beijing, China | FJ464769 | VP1 |
|  | GL361 | 2007 | Beijing, China | FJ464771 | VP1 |
|  | GL376 | 2007 | Beijing, China | FJ464776 | VP1 |
|  | GL377 | 2007 | Beijing, China | FJ464777 | VP1 |
|  | LZ50419 | 2006 | China | FJ586240 | VP1 |
|  | BCH133 | 2007 | Beijing, China | GU126461 | VP1 |
|  | BCH895 | 2008 | Beijing, China | GU126466 | VP1 |
|  | Pak3097 | 2009 | Pakistan | AB747247, AB747248 | VP1, genome |
|  | Pak1662 | 2009 | Pakistan | AB747239 | VP1 |
|  | Pak2100 | 2009 | Pakistan | AB747225 | VP1 |
|  | Pak3310 | 2009 | Pakistan | AB747211 | VP1 |
|  | Pak1729 | 2009 | Pakistan | AB747193 | VP1 |
|  | Afg1353 | 2009 | Afghanistan | AB747187 | VP1 |
|  | Afg879 | 2009 | Afghanistan | AB747177 | VP1 |
|  | Pak3379 | 2009 | Pakistan | AB747212 | VP1 |
|  | Pak1499 | 2009 | Pakistan | AB747190 | VP1 |
|  | Pak909 | 2009 | Pakistan | AB747188 | VP1 |
| SAFV-2 | UC6 | 2001 | USA | GU595289 | genome |
|  | S4 | 2005 | USA | JN652231 | genome |
|  | S14 | 2005 | USA | JN652232 | genome |
|  | S19 | 2005 | USA | JN652233 | genome |
|  | HTMV | 2005 | USA | NC010810 | genome |
|  | UC4 | 1999 | USA | EU604747 | VP1 |
|  | UC7 | 1999 | USA | EU604749 | VP1 |
|  | UC3 | 1999 | USA | EU604750 | VP1 |
|  | BCHU79 | 2009 | Beijing, China | GU126462, GU943518 | VP1, genome |
|  | FIN08-13B | 2008 | Finland | FJ374267 | VP1 |
|  | Pak971 | 2008 | Pakistan | FJ463601 | VP1 |
|  | BCHU353 | 2009 | Beijing, China | GU126464 | VP1 |
|  | D/VI2229/2004 | 2004 | Germany | EU681176 | genome |
|  | BR/118/2006 | 2006 | Germany | EU681177 | genome |
|  | Hu/CSF-03981/DEU/2007 | 2007 | Germany | JN209931 | 5'UTR, L, P1 |
|  | Hu/SIDS-347/DEU/2010 | 2010 | Germany | JN209932 | 5'UTR, L, P1, P2 |
|  | Nijmegen2008 | 2008 | Netherlands | FN999911 | genome |
|  | Finland2008 | 2008 | Finland | FR682076 | genome |
|  | Can112051-06 | 2006 | Canada | AM922293 | L, P1, P2, P3 |
|  | Pak2491 | 2009 | Pakistan | AB747203, AB747249 | VP1, genome |
|  | Pak3557 | 2009 | Pakistan | AB747215 | VP1 |
|  | Pak3430 | 2009 | Pakistan | AB747213 | VP1 |
|  | Pak3259 | 2009 | Pakistan | AB747244 | VP1 |
|  | Afg1308 | 2009 | Afghanistan | AB747182 | VP1 |
|  | QCW | 2011 | Australia | JX163901 | genome |
|  | QPID11-0004 | 2011 | Australia | JQ820266 | VP1 |
|  | QPID11-0002 | 2011 | Australia | JQ820264 | VP1 |
|  | QPID11-0005 | 2011 | Australia | JQ820267 | VP1 |
|  | QPID11-0001 | 2011 | Australia | JQ820263 | VP1 |
| SAFV-3 | JPN08-404 | 2008 | Japan | HQ902242 | genome |
|  | 07-Aichi10247 | 2007 | Aichi,Japan | AB542806 | VP1 |
|  | 07-Aichi10345 | 2007 | Aichi,Japan | AB542807 | VP1 |
|  | UC2 | 1999 | USA | EU604745 | VP1 |
|  | UC5 | 1999 | USA | EU604746 | VP1 |
|  | Pak2678 | 2007 | Pakistan | FJ463605 | VP1 |
|  | BCH350 | 2007 | Beijing,China | GU126465 | VP1 |
|  | BCHU115 | 2009 | Beijing,China | GU126463,GU943514 | VP1, genome |
|  | BCH1031 | 2008 | Beijing,China | GU126467,GU943513 | VP1, genome |
|  | Nijmegen2007 | 2007 | Netherlands | FM207487 | genome |
|  | D/VI2273/2004 | 2004 | Germany | EU681178 | genome |
|  | D/VI2223/2004 | 2004 | Germany | EU681179 | genome |
|  | NL1999-590 | 1998 | Netherlands | HM181996 | genome |
|  | NL2007-2686 | 2004 | Netherlands | HM181997 | genome |
|  | NL2007-2690 | 2004 | Netherlands | HM181998 | genome |
|  | NL2005-1035 | 2005 | Netherlands | HM181999 | genome |
|  | Penang | 2009 | Malaysia | HQ162476 | genome |
|  | Pak3641 | 2009 | Pakistan | AB747234, AB747250 | VP1, genome |
|  | Pak3103 | 2009 | Pakistan | AB747241 | VP1 |
|  | Pak3169 | 2009 | Pakistan | AB747207 | VP1 |
|  | Afg1280 | 2009 | Afghanistan | AB747185 | VP1 |
|  | Pak2678 | 2007 | Pakistan | FJ463605 | VP1 |
|  | Pak1851 | 2009 | Pakistan | AB747222 | VP1 |
|  | Pak3298 | 2009 | Pakistan | AB747210 | VP1 |
|  | Pak3253 | 2009 | Pakistan | AB747208 | VP1 |
|  | Afg1217 | 2009 | Afghanistan | AB747180 | VP1 |
|  | QPID11-0003 | 2011 | Australia | JQ820265 | VP1 |
| SAFV-4 | Pak3164 | 2009 | Pakistan | AB747206, AB747251 | VP1, genome |
|  | Pak962 | 2008 | Pakistan | FJ463603 | VP1 |
|  | Pak5842 | 2007 | Pakistan | FJ463606 | VP1 |
|  | Pak12 | 2008 | Pakistan | FJ463600 | VP1 |
|  | Pak2152 | 2009 | Pakistan | AB747229 | VP1 |
|  | Pak3635 | 2009 | Pakistan | AB747217 | VP1 |
|  | Pak2147 | 2009 | Pakistan | AB747197 | VP1 |
|  | Pak1619 | 2009 | Pakistan | AB747236 | VP1 |
|  | Pak3568 | 2009 | Pakistan | AB747232 | VP1 |
|  | Pak2339 | 2009 | Pakistan | AB747230 | VP1 |
|  | Pak3611 | 2009 | Pakistan | AB747216 | VP1 |
|  | Pak2123 | 2009 | Pakistan | AB747196 | VP1 |
|  | Afg1219 | 2009 | Afghanistan | AB747184 | VP1 |
| SAFV-5 | Pak5003 | 2007 | Pakistan | FJ463615 | genome |
|  | Pak5152 | 2007 | Pakistan | FJ463616 | genome |
|  | Pak3290 | 2009 | Pakistan | AB747209, AB747252 | VP1, genome |
|  | Pak2254 | 2009 | Pakistan | AB747243 | VP1 |
|  | Pak3633 | 2009 | Pakistan | AB747233 | VP1 |
|  | Pak1670 | 2009 | Pakistan | AB747221 | VP1 |
|  | Pak3125 | 2009 | Pakistan | AB747205 | VP1 |
|  | Pak2081 | 2009 | Pakistan | AB747195 | VP1 |
|  | Pak1200 | 2009 | Pakistan | AB747218 | VP1 |
| SAFV-6 | Pak6572 | 2007 | Pakistan | FJ463617 | genome |
|  | Pak1570 | 2009 | Pakistan | AB747219, AB747253 | VP1, genome |
|  | Pak1621 | 2009 | Pakistan | AB747237 | VP1 |
|  | Pak2396 | 2009 | Pakistan | AB747231 | VP1 |
|  | Pak2124 | 2009 | Pakistan | AB747227 | VP1 |
|  | Pak2026 | 2009 | Pakistan | AB747223 | VP1 |
|  | Afg1157 | 2009 | Afghanistan | AB747181 | VP1 |
|  | Pak1655 | 2009 | Pakistan | AB747220 | VP1 |
|  | Pak1709 | 2009 | Pakistan | AB747192 | VP1 |
| SAFV-7 | Pak1220 | 2009 | Pakistan | AB747189 | VP1, genome |
|  | Afg1449 | 2007 | Afghanistan | FJ463602 | VP1 |
|  | Afg1216 | 2009 | Afghanistan | AB747179 | VP1 |
|  | Pak2106 | 2009 | Pakistan | AB747242, AB747254 | VP1 |
|  | Pak1907 | 2009 | Pakistan | AB747240 | VP1 |
|  | Pak1628 | 2009 | Pakistan | AB747238 | VP1 |
|  | Pak2104 | 2009 | Pakistan | AB747226 | VP1 |
|  | Pak2335 | 2009 | Pakistan | AB747200 | VP1 |
|  | Pak1799 | 2009 | Pakistan | AB747194 | VP1 |
|  | Afg1314 | 2009 | Afghanistan | AB747186 | VP1 |
| SAFV-8 | Pak3486 | 2009 | Pakistan | AB747214, AB747255 | VP1, genome |
|  | Pak1141 | 2008 | Pakistan | FJ463604 | VP1 |
|  | Pak1666 | 2009 | Pakistan | AB747191 | VP1 |
|  | Pak2061 | 2009 | Pakistan | AB747224 | VP1 |
|  | Pak2496 | 2009 | Pakistan | AB747204 | VP1 |
|  | Pak2451 | 2009 | Pakistan | AB747202 | VP1 |
|  | Pak2229 | 2009 | Pakistan | AB747198 | VP1 |
| SAFV-9 | Pak2457 | 2009 | Pakistan | AB747256 | genome |
|  | Nig329 | 2007 | Nigeria | FJ997532 | VP1 |
| SAFV-10 | Pak2325 | 2009 | Pakistan | AB747199, AB747257 | VP1, genome |
| SAFV-11 | Pak2338 | 2009 | Pakistan | AB747201, AB747258 | VP1, genome |
|  | Pak3648 | 2009 | Pakistan | AB747235 | VP1 |
|  | Afg845 | 2009 | Afghanistan | AB747183 | VP1 |
|  | Pak2125 | 2009 | Pakistan | AB747228 | VP1 |
|  | Afg1155 | 2009 | Afghanistan | AB747178 | VP1 |
| Mengo virus | medium plague |  |  | L22089 | genome |
| TMEV | BeAn | 1957 | Brazil | M16020 | genome |
|  | DA | 1948 | Boston, USA | M20301 | genome |
|  | GDVII | 1937 | New York, USA | X56019 | genome |
| VHEV | V-1 | 1955 | Yakutsk, Russia | M80888, M94868, EU723237 | genome |
| EMCV | Ruckert |  |  | M81861 | genome |
